# Undernutrition and anaemia among Indian adolescents: role of dietary diversity and hygiene practices

**DOI:** 10.1017/jns.2023.19

**Published:** 2023-03-09

**Authors:** Mukesh Kumar, Pratap Chandra Mohanty

**Affiliations:** Department of Humanities and Social Sciences, Indian Institute of Technology Roorkee, Uttarakhand, India

**Keywords:** Adolescent undernutrition, Anaemia, Dietary diversity, Hygiene practices, India

## Abstract

In 2021, the Lancet Commission on adolescent nutrition highlighted the need to prioritise the elimination of adolescent malnutrition to tap the human capital potential and break the intergenerational malnutrition trap. The nutritional requirement during adolescence reaches its peak. The present study aims to appraise the prevalence of undernutrition (stunting and thinness) and anaemia among adolescents (10–19 years) in India and the role of socioeconomic, individual-level hygiene behaviour and dietary diversity in nutritional outcomes. We have used the nationally representative Comprehensive National Nutrition Survey (CNNS-2016–18) that covers children and adolescents (0–19 years) in India. The prevalence of stunting, anaemia and thinness among adolescents was 27⋅2, 28⋅5 and 24⋅1 %, respectively. Bivariate and multivariable logistic regression models were applied to estimate the likelihood of undernutrition. The likelihood of stunting was higher for late adolescence (OR 1⋅21, 95 % CI 1⋅15, 1⋅27), low dietary diversity (OR 1⋅37, 95 % CI 1⋅26, 1⋅49) and low hygiene behaviour compliance (OR 1⋅53, 95 % CI 1⋅42, 1⋅64). Adolescents from the poorest quintile were more likely to be stunted (OR 3⋅20, 95 % CI 2⋅94, 3⋅48), anaemic (OR 1⋅66, 95 % CI 1⋅47, 1⋅87) and thin (OR 1⋅68, 95 % CI 1⋅54, 1⋅82). We found that lower hygienic compliance was significantly associated with undernutrition and anaemia. Therefore, promoting hygienic practices should be emphasised to tackle undernutrition and anaemia. Furthermore, dietary diversity and poverty were strong predictors of stunting and thinness, therefore targeting the poor and focusing on improving dietary diversity should be the priority.

## Introduction

In 2021, the Lancet Commission on adolescent nutrition emphasises that ‘to end malnutrition *in all its forms*, interventions and investments are needed in later childhood and adolescence’. Adolescence (age 10–19 years) is an age of transition from childhood to adulthood. During this transitional phase, crucial biological, cognitive, psychosocial and physical changes take place, and 50 % of the adult weight and 20 % of overall height are acquired in adolescence. These changes are profoundly related to nutritional status, and they also affect nutritional requirements, resulting in highest nutritional requirements during adolescence in the life cycle. The Commission also highlighted that adolescence can be another (possibly last) window of opportunity for linear growth catch-up beyond the first 1000 days since birth^(1)^. Earlier, the 2016 Lancet Commission on adolescent health and wellbeing had raised concerns over the lack of adequate data on adolescent nutrition, particularly in developing countries. Therefore, it is still challenging for policy makers to design policies, strategies and nutritional interventions that can help to eliminate the adolescent malnutrition^(2)^.

Malnutrition is a complex phenomenon characterised by a multiplicity of factors ranging from the socioeconomic status to dietary intake, water, sanitation and hygiene (WASH) practices, and socio-political factors affecting policymaking^(3–5)^. Addressing the adolescent nutrition is fundamental to materialise the potential human capital gains and to break the intergenerational malnutrition trap. The World Health Organisation (WHO) has estimated that around 16 million adolescent girls, particularly in low- and middle-income countries (LMICs) with higher nutritional risks, become mothers every year^(1)^. In India, for socioeconomically vulnerable groups like disadvantaged caste and gender, education and other skills earned during adolescence are very crucial in determining their future socioeconomic status and labour market outcomes^(6)^. Furthermore, due to son preference, early-age marriage and negative intra-household biases in food distribution females are more vulnerable in India^(7)^.

Therefore, to understand the health, nutrition, hygiene practices and dietary intake-related aspects of adolescents in India, the Comprehensive National Nutrition Survey (CNNS, 2016–18) was implemented. The findings from CNNS (2016–18) data show that 27⋅2 and 28⋅5 % of the adolescents are stunted and anaemic, and only 11 % of adolescents have adequate dietary diversity. Recent studies on adolescent dietary diversity and nutrition have found that only 24 % of adolescents have a high-mix diet^(8)^ and inadequate dietary intake is significantly associated with poor nutritional outcomes among females in India^(9)^.

Furthermore, access to improved WASH services have been found to have a significant impact on child nutritional outcomes^(10–12)^. Chattopadhyay *et al.* find a significant positive association between WASH practices (beyond household level availability) and nutritional outcomes of adolescent girls in three states (Bihar, Chhattisgarh and Odisha) of India^(13)^. But due to lack of comprehensive and representative data on adolescent nutrition, the scope of earlier studies is a limited to specific region or gender^(8,9,13,14)^. Two recent studies on adolescent malnutrition^(15)^ and anaemia^(16)^ conducted using CNNS (2016–18) data shows substantial socioeconomic and regional disparities in adolescent malnutrition. However, none of these large-scale studies assessed the roles of dietary diversity and individual-level hygiene practices in adolescent malnutrition.

Therefore, the present study aims to bridge this critical gap, first, by analysing nationally representative data on adolescents in India that covers adolescent dietary intake and hygiene practices. Secondly, apart from the socioeconomic covariates (place of residence, wealth status, caste, religion, sex and education), we investigate the role of dietary diversity score (DDS) and two hygiene behaviour practice indexes in nutritional outcomes using the population-weighted CNNS (2016–18) data on adolescents in India. The findings of the study can serve as a basis for further in-depth analysis and evidence for policy formulation and programmes aiming to optimise the interventions which may enhance the nutritional outcomes of adolescents in India.

## Methods

### Study design

**Fig. 1.**
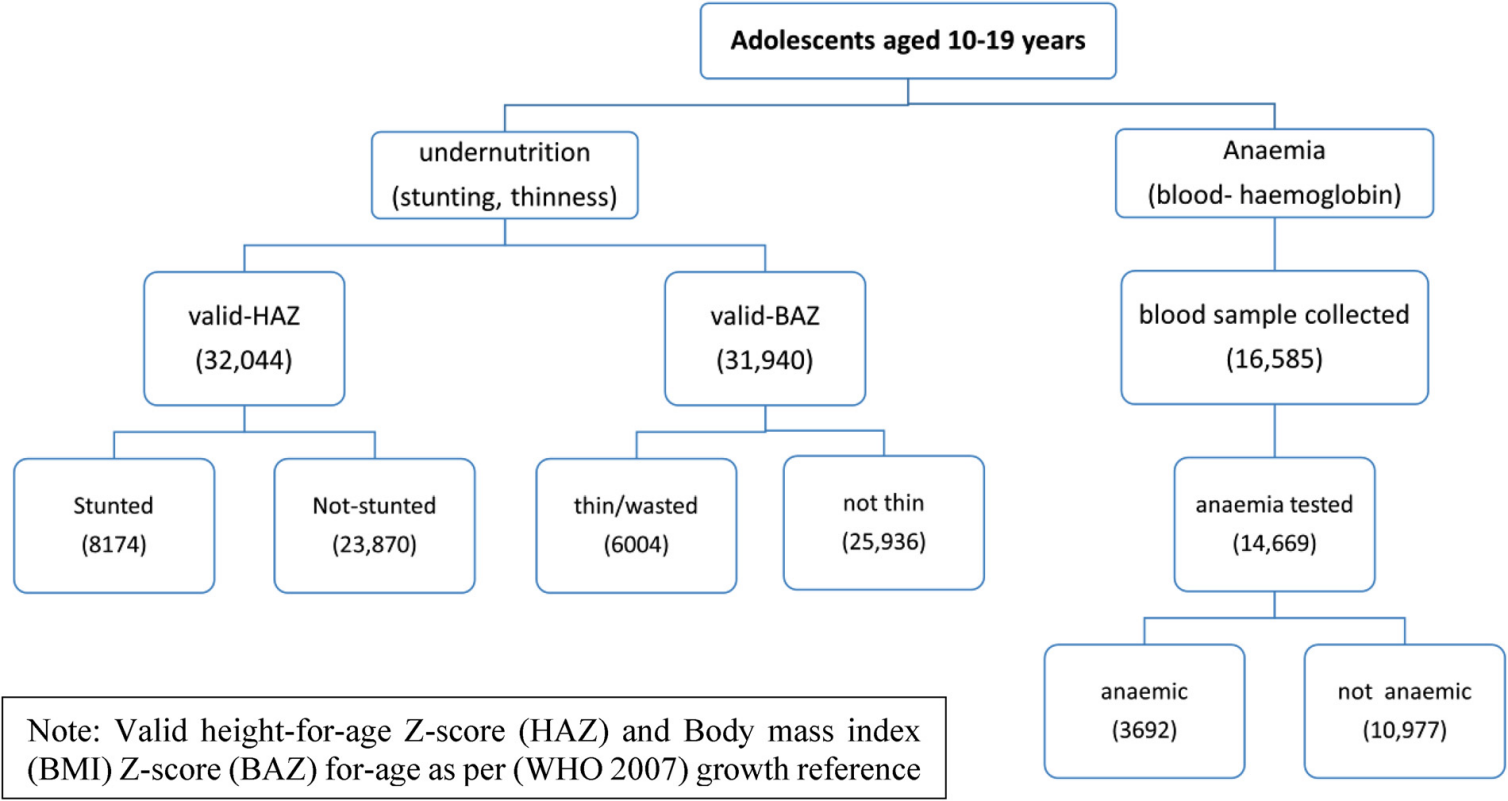
Flowchart of the sample included in the study.

The present study used a cross-section survey, CNNS 2016–18, data of a collaborative project of the Ministry of Health and Family Welfare (MoHFW), UNICEF and the Population Council of India. The survey was the largest micronutrient survey ever conducted globally. A multistage stratified cluster sampling design with probability proportionate to the size sample was used to attain a nationally representative sample of children as well as adolescents (below the age 19 years) in 29 states and the UT of Delhi^(17)^. To get information on different aspects of health, education, nutrition and demographic characteristics, separate schedules were designed with a common household schedule for children and early and late adolescents. Furthermore, the survey used gold standard cyanmethemoglobin methods to assess anaemia, micronutrient deficiencies and NCD for the first time in India. The unit of analysis in our study are the individuals aged 10–19 years. More details on sample selection and coverage of issues can be found from these sources^(17)^. Two analytical samples of undernutrition (stunting and thinness) and anaemia have been created for the final analysis Ethical standards disclosure (Fig. 1).

This study was conducted according to the guidelines laid down in the Declaration of Helsinki, and all the procedures involving human subjects/patients were approved by the Institutional Review Board (IRB), the Population Council (New York Office) and the Post Graduate Institute of Medical Research (PGIMR) Chandigarh, India^(17)^.

### Measuring undernutrition and anaemia

#### Malnutrition

Ideally, the definition of malnutrition involves both undernutrition and overnutrition (overweight/obesity). In developing countries, undernutrition and anaemia are still prevalent forms of malnutrition and they are used interchangeably. We have used the standard WHO's^(18)^ growth reference for adolescents aged 10–19 years to define malnutrition (undernutrition and anaemia).

#### Stunting and thinness

Stunting (low height-for-age) is defined as height-for-age *z*-score below −2 standard deviation (sd) from the (WHO 2007) adolescents growth reference. Similarly, a person aged 10–19 years is considered wasted/thin (low BMI-for-age) if his/her age-sex segregated BMI-for-age *z*-score is below −2sd^(19)^. Notably, due to rapid change in physical growth in puberty age, WHO has not defined the underweight for adolescents (aged 10–19 years)^(18)^.

#### Anaemia

The survey collected a blood sample of 8 ml from children aged 10–19 years to assess anaemia-related micronutrients and haemoglobin variants. For areas of higher than 1000 m altitude, haemoglobin concentrations were adjusted for altitude. Anaemia status is classified as per age-sex-specific criteria according to the WHO guidelines^(20)^. For ages 10–11 years and 12–14 years, a person is referred to as having anaemia if (s)he has a g/dl less than 11⋅5 and 12, respectively. For late adolescence (15–19 years), g/dl requirements are different for males (<13 g/dl) and females (<12 g/dl).

#### Outcome and predictor variables

Our primary-dependent variables are anthropometric measures (height- and weight-for-age) and anaemia. All three outcome variables are categorical and coded as ‘1’ for having anaemia or undernutrition and ‘0’ otherwise. The measurement and classification of the dependent variables is explained in the previous section. Based on the conceptual framework proposed by the Lancet Commission 2021, an extensive literature review and CNNS data availability, the study uses independent or predictor variables from the individual-, household- and community-level contexts.

#### Individual-level variables

As adolescence is characterised by age, which is on its own is an indicator of physical growth that determines the micro- and macronutrient requirement for the body. So, age is included as a dummy variable for early- and late adolescence (age 10–14 and 15–19 years). The literature provides evidence of gender biases in nutrition and food distribution within the household in India^(7)^. Hence, a dummy for the sex (male, female) of the respondent is also included in the determinants. The existing studies show significant positive impacts of hand hygiene on the health and nutritional outcomes of children in the Indian context^(21)^. Interestingly, the first-time questions on the hygiene behaviour of adolescents are included in the CNNS survey. The questions were asked using a dummy form i.e., when is it important to wash your hand? Based on ten questions, we constructed two hygiene indexes (general and critical) (Supplementary Table S1) based on their possible exposures to the pathogens. Six and four questions are considered for general and critical hygiene behaviour with equal weight. Both range from zero to two, where a score of zero, one and two implies no compliance, at least one, and two or more compliances, respectively. Education is another important contributor to health and nutrition^(22)^ via awareness and school-based supplementary nutrition programmes, i.e., Pradhan Mantri Poshan Shakti Nirman (PM-POSHAN, erstwhile Mid-Day Meal [MDM]) till early adolescence in India. We have included education as a dichotomous variable which provides us information about whether the child ever attended school or not. The availability of data on dietary intake and its frequency allows us to map dietary diversity and its role in determining nutritional outcomes. Based on the frequency of consumption of various food items: cereals, vegetables and fruits, eggs, meat, fish etc., a DDS is constructed using Food and Agriculture Organisation (FAO) guidelines for measuring adolescent dietary diversity (Supplementary Table S2)^(23)^. Following the literature, a cut-off score ≥4 is assumed as adequate dietary diversity^(23,24)^.

#### Household-level variables

Besides individual characteristics, the household environment also shapes nutritional outcomes. The household environment affects nutritional outcomes directly and indirectly via shaping individual behaviour and access to resources. A wealth index based on various household assets possession is used to assess the economic status, which divides the total population into five equal quintiles (poorest, poorer, middle, richer and richest). Caste and religion-based social stratification is an intrinsic feature of India's social system, which affects the socioeconomic status of individuals and households. CNNS data includes different categories of castes (Scheduled Caste, Scheduled Tribes, Other backward class and ‘Others’) and religions (Hindu, Muslim and Other religions) to which the household belongs. A dichotomous variable on whether mother attended school (yes, no) is also included in models because studies indicate that mother's education is an important predictor of child nutrition^(25)^.

#### Community-level or meso-environment variables

Though CNNS survey implemented a separate village questionnaire to capture community-level characteristics but partial data from this section is made available. These include the place of residence (rural–urban), states (Supplementary Table S3) and regions of the country based on agroecological and geographic features. There are six regions defined in CNNS (2016–18) survey from 29 states and the UT of Delhi (North, Central, East, North-East, West and South).

### Statistical analysis

To perform the statistical analysis, Stata 15⋅1^(26)^ software is used. Descriptive statistics for continuous variables are reported with mean and in the form of frequency distribution for categorical variables. *χ*^2^ test is applied to test the significance of the association. As our dependent variables are binary (stunted *v*. non-stunted, thin *v.* others, anaemic *v*. non-anaemic), logistic regression models have been used. Firstly, separate bivariate regressions have been applied for each category of predictors, and then multivariable models have been fitted to identify independent predictors. The independent variables which are statistically significant at the 5 % level in bivariate regressions are retained in multivariable models.

To take into consideration the CNNS multistage cluster sampling design, appropriate sampling weight has been applied. A more detailed description of the weighting procedure is available in CNNS report. The survey collected a blood sample for a subsample and therefore provided separate sampling weight for analysis of anthropometric (stunting/thinness) measures and biomarker (anaemia) samples. The odds ratios are reported at a 95 % confidence interval [CI] of statistical significance with a *P*-value <0⋅05 in two-tail.

## Results

The adolescent sample characteristics are presented in Table 1. The average age of adolescents in India is 14⋅4 years with a margin of ±2⋅8 sd. The age distribution of our sample is almost equal among early (51⋅8 %) and late adolescents (48⋅2). Half of the respondents are female, 75⋅3 % of the total adolescents reside in rural areas and 24⋅7 % are from urban settings. The groups are diverse in terms of both caste and religious belongingness. Twenty-two percent are from Scheduled Castes (SCs), 10⋅9 % are Scheduled Tribes (STs) and 41⋅7 % are of Other Backward Classes (OBCs). A majority of the respondents are Hindus (80⋅2 %), followed by Muslims (15⋅2 %) and ‘Others’ (4⋅5), including Sikh, Jain, Christian, etc. Among the sampled adolescents, 94⋅2 % reported that they had attended school, and 52⋅9 % of the mothers attended school/higher education. Surprisingly, only 11⋅4 % of adolescents have a DDS of 4 or greater. Based on a score of 0–6, 16 % of adolescents have zero compliance for general hygiene behaviour and only 36⋅9 % considered two or more hygiene practices important. Surprisingly, 16⋅4 % of the respondents think that it is not important to wash hands after; urine/passing stool, clean child faeces, touch pet waste or blow nose, which are taken as critical hygiene behaviour.

**Table 1.** Sample characteristics of adolescents (10–19 years) in India, CNNS 2016–18

Socioeconomic characteristics	Description	Percent (weighted)	Unweighted (*n*)	Total (unweighted)
Place of Residence	1 = Rural	75⋅3	19 606	35 830
	2 = Urban	24⋅7	16 224	
Sex	1 = Male	49⋅9	18 425	35 830
	2 = Female	50⋅1	17 405	
Age	Age*	14⋅4	35 830	35 830
	1 = 10–14 years	51⋅8	18 388	
	0 = 15–19 years	48⋅2	17 442	
Caste	1 = SC	22⋅5	6630	35 830
	2 = ST	10⋅9	6980	
	3 = OBC	41⋅7	11 419	
	4 = Others	24⋅9	10 801	
Religion	1 = Hindu	80⋅2	24 916	35 830
	2 = Muslim	15⋅2	4629	
	3 = Other Religion	4⋅5	6285	
Wealth index	1 = Poorest	20⋅0	3053	35 830
	2 = Poorer	20⋅0	4688	
	3 = Middle	20⋅0	6747	
	4 = Richer	20⋅0	9053	
	5 = Richest	20⋅0	12 289	
Mothers attended school	0 = No	52⋅9	13 321	35 830
	1 = Yes	47⋅1	22 509	
Children attending school	1 = Yes	94⋅2	34 625	35 830
	2 = No	5⋅8	1205	
General hygiene behaviour compliance	0 = No compliance	16⋅4	5256	35 830
	1 = At least one	46⋅7	17 083	
	2= Two or more	36⋅9	13 491	
Critical hygiene behaviour compliance	0 = No compliance	16⋅4	6516	35 830
	1 = At least one	66⋅6	23 967	
	2 = Two or more	16⋅9	5347	
Dietary diversity score ≥4	0 = No	88⋅6	30 905	35 824
	1 = Yes	11⋅4	4919	
National region	1 = North	22⋅9	8218	35 840
	2 = Central	11⋅2	4021	
	3 = East	14⋅5	5210	
	4 = North-East	11⋅0	3953	
	5 = West	16⋅0	5716	
	6 = South	24⋅3	8723	
Stunted	0 = No	72⋅8	23 870	32 044
	1 = Yes	27⋅2	8174	
Thinness/Wasting	0 = No	75⋅9	25 936	31 940
	1 = Yes	24⋅1	6004	
Anaemia	0 = No	71⋅5	10 977	14 669
	1 = Yes	28⋅5	3692	

* Value is mean.

### Prevalence of undernutrition and anaemia

Table 2 shows the socioeconomic and demographic breakdown of stunting, thinness and anaemia prevalence among adolescents in India. More than a quarter adolescent population of India is either stunted (27⋅2 %) or anaemic (28⋅5 %) or thin (24⋅1 %). Around 29⋅9 % of females are stunted, whereas the prevalence of stunting among male adolescents is 25⋅4 %. The prevalence of anaemia among females is 39⋅6 % compared with 17⋅7 % among males. In rural areas, 28⋅8, 29⋅1 and 25⋅3 % of adolescents are stunted, anaemic and thin, respectively. The rural–urban difference is lowest (2⋅2 %) in the case of anaemia compared with stunting (6⋅8 %) and thinness (4⋅8 %). Late adolescence is more prone to be stunted (29⋅2 %) and anaemic (33 %), whereas contrary to this, thinness is highest (27⋅4 %) in early adolescence. The highest prevalence of stunting (35⋅6 %) and anaemia (37⋅6 %) is among STs, and thinness is among OBCs (26⋅5 %). The prevalence is more pronounced among Muslims, with stunting (29⋅7 %), anaemia (31⋅8 %) and thinness (24⋅7 %).

**Table 2.** Prevalence of stunting, thinness and anaemia among adolescents (10–19 years) in India, CNNS 2016–18

Socioeconomic and demographic characteristics		Stunting (%)	*P*-value	Thinness (%)	*P*-value	Anaemia (%)	*P*-value
Sex	Male	25⋅4	0⋅527	29⋅4	<0⋅001	17⋅7	<0⋅001
	Female	28⋅9		18⋅9		39⋅6	
Residence	Rural	28⋅8	<0⋅001	25⋅3	<0⋅001	29⋅1	<0⋅001
	Urban	22⋅0		20⋅5		26⋅9	
Age	10–14 years	25⋅5	<0⋅001	27⋅4	<0⋅001	24⋅5	<0⋅001
	15–19 years	29⋅2		20⋅0		33⋅0	
Caste	SC	28⋅4	<0⋅001	22⋅9	<0⋅001	31⋅7	<0⋅001
	ST	35⋅6		22⋅2		37⋅6	
	OBC	26⋅7		26⋅5		23⋅8	
	Others	23⋅0		22⋅1		29⋅8	
Religion	Hindu	26⋅9	<0⋅001	24⋅4	<0⋅001	28⋅2	<0⋅001
	Muslim	29⋅7		24⋅7		31⋅8	
	Others	23⋅6		17⋅0		24⋅9	
Wealth index	Poorest	37⋅7	<0⋅001	27⋅2	<0⋅001	33⋅4	<0⋅001
	Poorer	30⋅8		26⋅6		29⋅3	
	Middle	28⋅6		26⋅0		28⋅3	
	Richer	21⋅9		22⋅2		28⋅7	
	Richest	15⋅9		18⋅2		23⋅2	
Mothers attended school	No	30⋅9	<0⋅001	25⋅6	<0⋅001	31⋅3	<0⋅001
	Yes	22⋅8		22⋅4		25⋅6	
Children attending school	Yes	26⋅5	<0⋅001	24⋅1	0⋅022	28⋅1	<0⋅001
	No	38⋅2		24⋅6		38⋅3	
General hygiene behaviour compliance	No compliance	33⋅5	<0⋅001	24⋅2	<0⋅001	29⋅9	<0⋅001
	At least one	26⋅8		25⋅7		26⋅0	
	Two or more	24⋅8		22⋅1		31⋅2	
Critical hygiene behaviour compliance	No compliance	32⋅9	<0⋅001	25⋅1	0⋅953	31⋅5	0⋅155
	At least one	25⋅9		23⋅9		28⋅0	
	Two or more	26⋅5		23⋅9		27⋅4	
Dietary diversity score ≥4	No	27⋅8	<0⋅001	24⋅5	<0⋅001	28⋅7	<0⋅001
	Yes	22⋅0		21⋅0		27⋅4	
National region	North	18⋅1	<0⋅001	24⋅1	<0⋅001	25⋅1	<0⋅001
	Central	28⋅6		24⋅5		28⋅9	
	East	31⋅3		23⋅9		34⋅2	
	North-East	29⋅0		26⋅7		30⋅0	
	West	22⋅1		23⋅0		19⋅6	
	South	39⋅6		17⋅0		33⋅5	
Total	India	**27⋅2**		**24⋅1**		**28⋅5**	

We find that undernutrition prevalence is significantly lower among the adolescents whose mothers attended school. The prevalence of stunting is 11⋅7 percentage points (pps) higher among the adolescents who do not attend school. In case of thinness, the prevalence does not differ significantly between school attendees (24⋅1 %) and non-attenders (24⋅6 %). Those adolescents who consider maintaining hygiene is important have a significantly lower prevalence of stunting, anaemia and thinness (Table 2). Respondents having a DDS <4 is significantly associated with a higher prevalence of undernutrition and anaemia. The prevalence of stunting (31⋅3 %), anaemia (34⋅3 %) and thinness (23⋅9 %) are highest in the Eastern region, which comprises larger and relatively poorer states (Bihar, Jharkhand, Odisha and West Bengal).

### Predictors of undernutrition and anaemia

To identify the likelihood of being undernourished or anaemic, results of bivariate logistic regressions are presented in Table 3. Late adolescent age respondents are more likely to be stunted and anaemic but less likely to be thin. Female had higher odds of being stunted and anaemic compared with males and lower likelihood of thinness. Among all other predictors classified under individual correlates, dietary diversity, hygiene behaviour and education were found to have a statistically significant association with stunting and anaemia prevalence. Adolescents of the social group ST have higher odds of stunting and anaemia compared with ‘Others’. The wealth index is another strongly associated factor with undernourishment, and the adolescents in the poorest wealth quintile are more likely to be stunted.

**Table 3.** Bivariate logistic regression results for unadjusted odds ratios for stunting, anaemia and thinness among adolescents (10–19 years) in India, CNNS 2016–18

Socioeconomic and demographic characteristics		Stunting (*n* 32 044)			Anaemia (*n* 14 669)			Thinness (*n* 31 940)		
		Odds ratio	*P*-value	95 %, [CI]	Odds ratio	*P*-value	95 %, [CI]	Odds ratio	*P*-value	95 %, [CI]
Age	10–14 years®									
	15–19 years	1⋅21	0⋅000	1⋅15–1⋅27	1⋅52	0⋅000	1⋅41–1⋅63	0⋅66	0⋅000	0⋅63–0⋅70
Sex	Male®									
	Female	1⋅19	0⋅000	1⋅14–1⋅25	3⋅06	0⋅000	2⋅84–3⋅31	0⋅56	0⋅000	0⋅53–0⋅59
Residence	Rural	1⋅43	0⋅000	1⋅35–1⋅52	1⋅11	0⋅012	1⋅02–1⋅21	1⋅32	0⋅000	1⋅24–1⋅4
	Urban®									
Caste	SC	1⋅32	0⋅000	1⋅23–1⋅42	1⋅09	0⋅097	0⋅98–1⋅21	1⋅05	0⋅227	0⋅97–1⋅13
	ST	1⋅85	0⋅000	1⋅70–2⋅02	1⋅42	0⋅000	1⋅25–1⋅61	1⋅01	0⋅868	0⋅92–1⋅11
	OBC	1⋅22	0⋅000	1⋅14–1⋅3	0⋅73	0⋅000	0⋅67–0⋅8	1⋅27	0⋅000	1⋅19–1⋅35
	Others®			-			-			-
Religion	Hindu	1⋅19	0⋅006	1⋅05–1⋅35	1⋅18	0⋅072	0⋅99–1⋅42	1⋅57	0⋅000	1⋅36–1⋅81
	Muslim	1⋅37	0⋅000	1⋅2–1⋅57	1⋅41	0⋅001	1⋅15–1⋅72	1⋅60	0⋅000	1⋅37–1⋅86
	Others®			-			-			-
Wealth index	Poorest	3⋅20	0⋅000	2⋅94–3⋅48	1⋅66	0⋅000	1⋅47–1⋅87	1⋅68	0⋅000	1⋅54–1⋅82
	Poorer	2⋅34	0⋅000	2⋅15–2⋅55	1⋅37	0⋅000	1⋅22–1⋅54	1⋅63	0⋅000	1⋅5–1⋅78
	Middle	2⋅11	0⋅000	1⋅94–2⋅3	1⋅31	0⋅000	1⋅16–1⋅47	1⋅58	0⋅000	1⋅45–1⋅72
	Richer	1⋅48	0⋅000	1⋅35–1⋅62	1⋅33	0⋅000	1⋅19–1⋅5	1⋅28	0⋅000	1⋅17–1⋅4
	Richest®			-			-			-
Mothers attended school	No	1⋅51	0⋅000	1⋅44–1⋅59	1⋅32	0⋅000	1⋅23–1⋅42	1⋅20	0⋅000	1⋅14–1⋅26
	Yes®			-			-			-
Children attending school	Yes®			-			-			-
	No	1⋅72	0⋅000	1⋅56–1⋅89	1⋅59	0⋅000	1⋅35–1⋅88	1⋅03	0⋅633	0⋅92–1⋅15
General hygiene behaviour compliance	No compliance	1⋅53	0⋅000	1⋅42–1⋅64	0⋅94	0⋅262	0⋅85–1⋅05	1⋅13	0⋅002	1⋅04–1⋅22
	At least one	1⋅11	0⋅000	1⋅05–1⋅18	0⋅78	0⋅000	0⋅72–0⋅84	1⋅22	0⋅000	1⋅15–1⋅29
	Two or more®			-			-			-
Critical hygiene behaviour compliance	No compliance	1⋅36	0⋅000	1⋅25–1⋅48	1⋅22	0⋅002	1⋅08–1⋅38	1⋅07	0⋅146	0⋅98–1⋅17
	At least one	0⋅97	0⋅380	0⋅91–1⋅04	1⋅03	0⋅541	0⋅93–1⋅14	1⋅00	0⋅937	0⋅94–1⋅08
	Two or more®			-			-			-
Dietary diversity score ≥4	No	1⋅37	0⋅000	1⋅26–1⋅49	1⋅06	0⋅281	0⋅95–1⋅19	1⋅22	0⋅000	1⋅12–1⋅32
	Yes®			-			-			-
National region	North®			-						-
	Central	1⋅82	0⋅000	1⋅67–1⋅98	1⋅22	0⋅002	1⋅08–1⋅37	1⋅02	0⋅557	0⋅94–1⋅11
	East	2⋅07	0⋅000	1⋅89–2⋅26	1⋅55	0⋅000	1⋅37–1⋅76	0⋅99	0⋅853	0⋅91–1⋅08
	North-East	1⋅86	0⋅000	1⋅67–2⋅06	1⋅28	0⋅001	1⋅11–1⋅48	1⋅15	0⋅007	1⋅04–1⋅27
	West	1⋅29	0⋅000	1⋅16–1⋅43	0⋅73	0⋅000	0⋅63–0⋅85	0⋅94	0⋅230	0⋅86–1⋅04
	South	2⋅98	0⋅000	2⋅56–3⋅45	1⋅51	0⋅000	1⋅21–1⋅88	0⋅65	0⋅000	0⋅54–0⋅77

Table 4 shows the estimates of multivariable logistic regressions applied to identify the statistical significance of individual variables after mutually adjusting for other confounders. Even after controlling for other correlates, females have a higher likelihood of stunting (OR 1⋅19; 95 % CI 1⋅14, 1⋅26) and anaemia (OR 3⋅13; 95 % CI 2⋅89, 3⋅40). In multivariable models, the odds (OR 0⋅93; 95 % CI 0⋅86, 0⋅99) of stunting become lower in rural areas compared with urban areas. In the case of thinness, rural adolescents are more likely to be thin (OR 1⋅12; 95 % CI 1⋅04, 1⋅21). Though magnitude lowers, the likelihood of stunting (OR 1⋅43; 95 % CI 1⋅30, 1⋅58) and anaemia (OR 1⋅62; 95 % CI 1⋅40, 1⋅87) remains higher among STs. Compared with ‘Others’ religious groups, Muslim adolescents are more likely to be stunted (OR 1⋅37; 95 % CI 1⋅18, 1⋅59), anaemic (OR 1⋅46; 95 % CI 1⋅17, 1⋅83) and thin (OR 1⋅44; 95 % CI 1⋅23, 1⋅70). Economic status remains the strongest predictor of stunting and thinness, even after controlling for social and demographic covariates. Being a member of the poorest wealth quintile households, the likelihood of stunting approximately three times (OR 2⋅83; 95 % CI 2⋅54, 3⋅17), anaemia (OR 1⋅66; 95 % CI 0⋅99, 1⋅35) and thinness (OR 1⋅83; 95 % CI 1⋅63, 2⋅04). Adolescents with a lower score on hygiene practices have higher probabilities of stunting (OR 1⋅20; 95 % CI 1⋅08, 1⋅33) and anaemia (OR 1⋅24; 95 % CI 1⋅03, 1⋅48). A DDS of <4 predicts a higher likelihood of stunting (OR 1⋅14; 95 % CI 1⋅05, 1⋅24) (Table 4). The results of multivariable models reinforce the finding of bivariate models even after controlling for various socioeconomic and household correlates.

**Table 4.** Multivariable logistic regression results (adjusted odds ratios) for stunting, anaemia and thinness among adolescents (10–19 years) in India, CNNS 2016–18

Socioeconomic and demographic characteristics		Stunting (*n* 32 044)			Anaemia (*n* 14 669)			Thinness (*n* 31 940)		
		Odds ratio	*P*-value	95 %, [CI]	Odds ratio	*P*-value	95 %, [CI]	Odds ratio	*P*-value	95 %, [CI]
Age	10–14 years®									
	15–19 years	1⋅26	0⋅000	1⋅20–1⋅33	1⋅53	0⋅000	1⋅42–1⋅66	0⋅67	0⋅000	0⋅63–0⋅71
Sex	Male®									
	Female	1⋅19	0⋅000	1⋅14–1⋅26	3⋅13	0⋅000	2⋅89–3⋅40	0⋅56	0⋅000	0⋅53–0⋅59
Residence	Rural	0⋅93	0⋅035	0⋅86–0⋅99	-	-	-	1⋅12	0⋅002	1⋅04–1⋅21
	Urban®				-	-	-			
Caste	SC	1⋅26	0⋅000	1⋅16–1⋅36	1⋅22	0⋅001	1⋅08–1⋅37	0⋅97	0⋅499	0⋅89–1⋅06
	ST	1⋅43	0⋅000	1⋅30–1⋅58	1⋅62	0⋅000	1⋅40–1⋅87	0⋅88	0⋅012	0⋅79–0⋅97
	OBC	1⋅22	0⋅000	1⋅14–1⋅31	0⋅80	0⋅000	0⋅72–0⋅89	1⋅23	0⋅000	1⋅15–1⋅32
	Others®									
Religion	Hindu	1⋅06	0⋅409	0⋅92–1⋅21	1⋅25	0⋅026	1⋅03–1⋅53	1⋅29	0⋅001	1⋅12–1⋅50
	Muslim	1⋅37	0⋅000	1⋅18–1⋅59	1⋅46	0⋅001	1⋅17–1⋅83	1⋅44	0⋅000	1⋅23–1⋅70
	Others®									
Wealth index	Poorest	2⋅83	0⋅000	2⋅54–3⋅17	1⋅16	0⋅064	0⋅99–1⋅35	1⋅83	0⋅000	1⋅63–2⋅04
	Poorer	2⋅10	0⋅000	1⋅89–2⋅33	1⋅04	0⋅595	0⋅90–1⋅2	1⋅74	0⋅000	1⋅57–1⋅94
	Middle	1⋅95	0⋅000	1⋅77–2⋅15	1⋅05	0⋅483	0⋅92–1⋅2	1⋅67	0⋅000	1⋅52–1⋅84
	Richer	1⋅39	0⋅000	1⋅27–1⋅53	1⋅16	0⋅019	1⋅03–1⋅32	1⋅31	0⋅000	1⋅2–1⋅44
	Richest®									
Mothers attended school	No	1⋅07	0⋅020	1⋅01–1⋅14	1⋅14	0⋅005	1⋅04–1⋅24	1⋅04	0⋅277	0⋅97–1⋅10
	Yes®									
Children attending school	Yes®									
	No	1⋅21	0⋅000	1⋅09–1⋅34	1⋅24	0⋅021	1⋅03–1⋅48	0⋅99	0⋅890	0⋅88–1⋅11
General hygiene behaviour compliance	No compliance	1⋅19	0⋅000	1⋅09–1⋅31	0⋅91	0⋅154	0⋅8–1⋅04	1⋅02	0⋅669	0⋅93–1⋅12
	At least one	1⋅11	0⋅000	1⋅05–1⋅18	1⋅00	0⋅986	0⋅92–1⋅09	1⋅05	0⋅097	0⋅99–1⋅12
	Two or more®									
Critical hygiene behaviour compliance	No compliance	1⋅20	0⋅001	1⋅08–1⋅33	–	–	–	–	–	–
	At least one	0⋅96	0⋅297	0⋅9–1⋅03	–	–	–	–	–	–
	Two or more®				–	–	–	–	–	–
Dietary diversity score ≥4	No	1⋅14	0⋅003	1⋅05–1⋅24	–	–	–	1⋅05	0⋅239	0⋅97–1⋅15
	Yes®				–	–	–			
National region	North®									
	Central	1⋅28	0⋅000	1⋅17–1⋅4	1⋅17	0⋅021	1⋅02–1⋅34	0⋅84	0⋅000	0⋅77–0⋅91
	East	1⋅45	0⋅000	1⋅32–1⋅6	1⋅53	0⋅000	1⋅33–1⋅76	0⋅77	0⋅000	0⋅7–0⋅84
	North-East	1⋅58	0⋅000	1⋅39–1⋅78	1⋅44	0⋅000	1⋅21–1⋅71	1⋅18	0⋅005	1⋅05–1⋅33
	West	1⋅24	0⋅000	1⋅11–1⋅38	0⋅80	0⋅004	0⋅68–0⋅93	0⋅91	0⋅057	0⋅82–1⋅00
	South	1⋅92	0⋅000	1⋅61–2⋅28	1⋅64	0⋅000	1⋅27–2⋅11	0⋅57	0⋅000	0⋅46–0⋅69

## Discussion

The present study assesses the prevalence and predictors associated with undernutrition and anaemia among adolescents in India. Besides socioeconomic factors, we have also examined the role of perceived hand hygiene behaviour and dietary diversity in nutritional outcomes. The study has found that stunting (29⋅9 %) and anaemia (39⋅6 %) are more prevalent among females^(16)^. Females are relatively more disadvantaged (2⋅23 times) in terms of anaemia compared with stunting. A similar degree of relative disadvantage for the female child (age 0–9 years) in case of anaemia has been reported in CNNS 2016–18 report. This indicates persistent gender disadvantage in terms of anaemia prevalence. As the children reach adolescence maturity, the gap in anaemia prevalence between males and females starts rising and reaches its peak at the age of 18–19 years. Furthermore, this gap remains persistent during the life course^(27–29)^. At the same time, the prevalence of thinness is significantly lower (10 % points) among females. It may have multiple interpretations as the mean age at menarche in India is 13⋅8 years^(30)^, and menarche is associated with rapid weight gain in late adolescence^(31)^. This is also reflected in the age-sex disaggregated analysis of thinness, where the difference in thinness among males and females is 8 % points in early adolescence (10–14 years) which later extends up to13 % points in later adolescence (15–19 years), followed by the start of menarche. Due to these uneven differences in weight changes, it is difficult to determine the underweight criterion for adolescence age^(18)^. Noteworthy, after reaching adulthood and entering childbearing age, the pattern of thinness prevalence is reversed, and women become more vulnerable to being underweight^(29)^.

In terms of nutritional outcomes, there already exists large rural–urban disparities among children and adults in India^(32)^. Similarly, we also observe a substantially higher prevalence of stunting (6⋅8 pp), thinness (5 pp) and anaemia (2⋅2 pp) among rural adolescents. The potential mechanism can be the unaffordability of nutritious diet (only 24–37 % can afford a nutritious diet) in the rural area^(33)^ and the widespread lack of access to and utilisation of WASH facilities^(34)^. The present study finds an upward shift in stunting (4 %-point increase) and anaemia (9 % points increase) after early adolescence. The underlying reasons may be the increased intensity of physical and cognitive activities in late adolescence, which further deepens the vulnerabilities of existing undernourished adolescents.

There are two major supplementary nutrition programmes in India: Integrated Child Development Services (ICDS) and PM-POSHAN (erstwhile MDM). ICDS provides supplementary nutrition and health check-up services for children (age 0–6 years), adolescent girls, pregnant women and lactating mothers^(35)^. Through PM-POSHAN hot cooked meal is provided in government and government-aided schools from class (I–VIII) to improve child nutritional and educational outcomes. Impact evaluation studies show that the beneficiaries of MDM have better nutrition and cognitive outcomes^(36,37)^. Our study has also found that the likelihood of being anaemic and stunted is higher among those adolescents who do not attend school.

Low dietary diversity makes adolescents more vulnerable to undernutrition^(14,38)^, and we also find that lower DDS is significantly associated with poor nutritional outcomes. Mersha *et al.* find 2⋅47 times higher likelihood of undernutrition among the adolescents having inadequate DD and food insecurity^(39)^. In India, by making more diverse and nutritious, PM-POSHAN can help to improve the nutritional outcomes, at least, in early adolescence as the mean age at class (VIII) is 15 years up to which cooked meal is served in the schools. Furthermore, on experimental basis, PM-POSHAN can be extended to higher classes to assess the benefits of such a scheme in late adolescence.

Wealth-based poverty is another factor that significantly determines nutritional outcomes in India and our findings are also consistent with the existing studies^(40,41)^. In a long-term, large-scale experimental setting, ‘Targeting the Ultra Poor’ (TUP) in West Bengal^(42)^ has found a positive and durable effect of transfer on food security, consumption, income and health. This kind of policy intervention should be adopted, which simultaneously improves health and nutritional outcomes and economic status.

Among other predictors, we find that better hygiene and sanitation awareness contributes significantly to nutritional outcomes. Earlier studies in the Indian context have found that improved Water Sanitation and Hygiene (WASH) services are positively associated with child nutritional outcomes^(43,44)^. But these studies are limited to *access and availability* and do not touch on the behavioural aspects. We have analysed the hygiene practices among adolescents using two indexes. The finding is worrisome and reveals that one-sixth of the adolescents do not consider hygiene practices important at all and are more vulnerable to being undernourished. Chattopadhyay *et al.* in their study on adolescent girls in Bihar, Chhattisgarh and Odisha also found that handwashing without soap after defecation is associated with increased stunting and thinness. Our findings are consistent with the study conducted in Ethiopia that finds that handwashing before eating lowers the odds (0⋅67) of stunting^(38)^. This seems very much relevant in the present context because, based on *ownership* of toilets, the present government has declared India as open defecation-free (ODF). And now, practising hygienic behaviour is another important area that requires attention^(45)^. In this direction, the Swachh Bharat Mission-Gramin (SBM-G) has adopted sanitation behaviour change communication to nudge and mobilise communities to adopt sustainable hygiene and sanitation practices^(46)^. A recent study on rational habit formation in the context of handwashing in rural India proposes that monitoring and incentivising results in persistent handwashing, which ultimately improves child health and nutrition outcomes^(21)^.

The existing literature documents the poor health, education and nutrition outcomes among historically marginalised social groups^(47)^. Our study also has also found that even after controlling for demographic and other covariates, adolescents of disadvantaged sections are more vulnerable to undernutrition and anaemia.

## Conclusion

The present study examines the prevalence and predictors of adolescent undernutrition and anaemia in India using CNNS 2016–18 data. Among an array of socioeconomic and demographic factors, we find that the likelihood of stunting and anaemia is significantly higher in late adolescence (age 15–19 years). Females are three times more likely to have anaemia compared with males. We also find that dietary diversity and hygiene behaviour are significant predictors of stunting and anaemia among adolescents in India. After conditioning on socioeconomic characteristics, the magnitude of dietary diversity and hygiene behaviour decreases but remains statistically significant. The likelihood of stunting remains 14 and 20 % higher among adolescents having inadequate dietary diversity and poor hygiene practices compliance. Maternal and adolescent's education are also significant predictors of adolescent nutrition status. The mechanism through which education can affect nutrition is that educated mothers and children may have more aware food choices and a better understanding of the significance of hygiene practices.

During adolescence (age 10–19 years), rapid and crucial physical, mental and psychosocial changes occur. Therefore, adolescent health and nutrition require special attention to break the vicious cycle of the intergenerational malnutrition trap. Apart from socioeconomic predictors, the present study has identified the role of hygiene and sanitation behaviour and dietary diversity in adolescent nutrition and anaemia status in India.

### Limitation

Despite several strengths and salient findings, we accept to have some limitations of the study. First, the DDS does not indicate the quantity of food consumed as the questions are asked on the frequency and the type of food items consumed. Dietary intake may be affected by seasons, and some food items can be available in large quantities and at low cost for limited periods. Second, disaggregated data on years of schooling is not made available till now in the CNNS dataset. It is important to have these data to assess the impact/role of school-based feeding programmes PM-POSHAN on adolescents’ dietary diversity, anaemia and nutritional outcomes. Third, puberty results in rapid weight gain among females. Therefore, we suspect that the uneven prevalence of thinness is affected by this factor, but due to the lack of data on age at menarche, we cannot factor out this. Age at menarche was asked in the survey, but the data have not been released yet. Fourth, due to the cross-sectional nature of the data, we cannot claim the findings as a causal relation. The survey was designed to be representative at the state level, therefore, further disaggregated analysis is not possible.

### Policy implications

Apart from the policy relevance of targeting socioeconomically vulnerable groups, the present study has two key takeaways. First, after achieving the household toilet ownership-based universal ODF target, our study underlies the indicative importance of the behavioural aspect of hygienic practices on nutritional outcomes^(46,48)^. More focused behaviour modification approaches highlighting the positive impacts of practising sanitation and hygiene should be emphasised in the Swachh Bharat Mission (SBM)^(21)^.

Second, the CNNS survey was completed well before the outbreak of the COVID-19 pandemic, and the crisis increased the risk of food-and-nutrition insecurity. Therefore, more targeted and direct nutrition intervention should be implemented. This can be done by expanding PM-POSHAN to higher classes and making it more nutritious and diverse. POSHAN 2⋅0 (renamed and extended provisions of ICDS), which makes provisions for nutritional and health requirements of out-of-school adolescent girls (age 14–18 years), is a very important step in this direction^(35)^. However, despite the disruption in delivery mechanisms of school-based feeding programmes and estimated rise in hunger and undernutrition, the budget allocation for POSHAN 2⋅0 has remained stagnant over the last 2 years (2021–22 and 2022–23)^(49)^.

## Supplementary material

For supplementary material accompanying this paper visit https://doi.org/10.1017/jns.2023.19. click here to view supplementary material
