# Supplementary material for: Undernutrition and anaemia among Indian adolescents: role of dietary diversity and hygiene practices
Source: J Nutr Sci. 2023 Mar 9;12:e33. doi: 10.1017/jns.2023.19 (PMC10052428; doi:10.1017/jns.2023.19)
Supplement: Supplementary file 1 [file S2048679023000198sup001.docx]

**S1: Respondent’s perceived importance of washing hand after following activities and construction of hygiene index.**

| **Sr. no** | **When it is important to wash hand?** | **Response** | **Final** |
| --- | --- | --- | --- |
| 1. | Before cooking/handling food | Yes 1, no 0 | General  Hygiene  Practices |
| 2. | Before eating food | Yes 1, no 0 |  |
| 3. | Before feeding an infant/younger sibling | Yes 1, no 0 |  |
| 4. | Before preparing food | Yes 1, no 0 |  |
| 5. | After play or work outside | Yes 1, no 0 |  |
| 6. | After meal | Yes 1, no 0 |  |
| **#** | **Max. general hygiene score** | **-** | **6** |
| 7. | After passing urine/stool | Yes 1, no 0 | Critical  Hygiene  Practices |
| 8. | After cleaning a child’s faeces | Yes 1, no 0 |  |
| 9. | After touching pets or handling animals and their waste | Yes 1, no 0 |  |
| 10. | After blowing nose or coughing | Yes 1, no 0 |  |
| **#** | **Max. critical hygiene score** | **-** | **4** |

Source: Authors’ estimation from CNNS 2016-18 unit-level data.

**S2: Measurement of dietary diversity based on frequency of food item intake.**

| **Sr. no** | **Food item** | **No. of days consumed in a week** | **DD category** | **Dietary Diversity Score** |
| --- | --- | --- | --- | --- |
| 1. | Cereals | 7=1, 0 otherwise | Starchy staples | 1 if sr. no 1 or 2=1, 0 otherwise |
| 2. | Roots and tubers | 7=1, 0 otherwise |  |  |
| 3. | Green leafy vegetables | 7=1, 0 otherwise | Dark green leafy vegetables | 1 if sr. no 3=1, 0 otherwise |
| 4. | Other vegetables | 7=1, 0 otherwise | Fruit and vegetables | 1 if sr. no 4 or 5=1, 0otherwise |
| 5. | Fruits | 7=1, 0 otherwise |  |  |
| 6. | Pulses or beans | 7=1, 0 otherwise | Nuts and oilseeds | 1 if sr. no 6 or 7=1, 0 otherwise |
| 7. | Nuts and oilseed | 7=1, 0 otherwise |  |  |
| 8. | Eggs | 7=1, 0 otherwise | Egg | 1 if sr. no 8=1, 0 otherwise |
| 9. | Fish | 7=1, 0 otherwise | Meat and fish | 1 if sr. no 9 or 10=1, 0 otherwise |
| 10. | Chicken or meat | 7=1, 0 otherwise |  |  |
| 11. | Milk or milk products | 7=1, 0 otherwise | Milk and milk product | 1 if sr. no 11=1, 0 otherwise |
| 12. | Total (Maximum) | 11 | - | 7 (Max.) |

Source: Authors’ estimation from CNNS 2016-18 unit-level data using FAO dietary diversity guidelines.

**S3: State level Prevalence of stunting, anaemia, and thinness among adolescents (10-19 years) in India, Comprehensive National Nutrition Survey 2016-18**

| states | Stunting^*^ (%) | Anaemia^*^ (%) | Thinness^*^ (%) |
| --- | --- | --- | --- |
| Jammu and Kashmir | 18.55 | 16.62 | 13.18 |
| Himachal Pradesh | 20.01 | 15.58 | 31.12 |
| Punjab | 16.16 | 25.52 | 18.02 |
| Uttarakhand | 21.07 | 15.63 | 15.37 |
| Haryana | 16.03 | 29.82 | 20.68 |
| NCT of Delhi | 21.1 | 30.01 | 21.1 |
| Rajasthan | 17.91 | 26.14 | 29.07 |
| Uttar Pradesh | 28.34 | 31.81 | 22.13 |
| Bihar | 32.15 | 27.2 | 22.89 |
| Sikkim | 23.74 | 26.1 | 9.9 |
| Arunachal Pradesh | 29.96 | 26.17 | 7.86 |
| Nagaland | 41.76 | 9.01 | 10.54 |
| Manipur | 26.34 | 10.26 | 6.18 |
| Mizoram | 29.05 | 17.94 | 6.37 |
| Tripura | 39.62 | 41.37 | 16.55 |
| Meghalaya | 45.36 | 31.62 | 6.77 |
| Assam | 40.86 | 36.96 | 19.81 |
| West Bengal | 29.85 | 45.57 | 25.39 |
| Jharkhand | 33.94 | 34.07 | 28.28 |
| Odisha | 27.59 | 29.09 | 18.53 |
| Chhattisgarh | 25.92 | 31.44 | 18.13 |
| Madhya Pradesh | 29.83 | 21.18 | 31.83 |
| Gujarat | 25.44 | 33.86 | 30.29 |
| Maharashtra | 31.18 | 27.91 | 24.65 |
| Andhra Pradesh | 25.58 | 21.28 | 19.69 |
| Karnataka | 27.77 | 17.09 | 27.2 |
| Goa | 16.95 | 13.49 | 21.68 |
| Kerala | 14.22 | 9.28 | 20.08 |
| Tamil Nadu | 18.88 | 15.2 | 20.77 |
| Telangana | 20.42 | 32.42 | 28.82 |
| **TOTAL** | **27.15** | **28.51** | **24.11** |

* Figures are weighted

Source: Authors’ estimation from CNNS 2016-18 unit-level data.
